# Detecting emotional disorder with eye movement features in sports watching

**DOI:** 10.3389/fneur.2025.1562785

**Published:** 2025-04-29

**Authors:** Wei Qiang, Lin Yang, Xucheng Zhang, Na Liu, Yanyong Wang, Jipeng Zhang, Yixin Long, Weiwei Xu, Wei Sun

**Affiliations:** ^1^Institute of Software, Chinese Academy of Sciences, Beijing, China; ^2^School of Computer Science and Technology, University of Chinese Academy of Sciences, Beijing, China; ^3^Nursing Department, The First Hospital of Hebei Medical University, Hebei, China; ^4^Health Service Department of the Guard Bureau of the Joint Staff Department, Beijing, China; ^5^School of Medical Humanities, Capital Medical University, Beijing, China

**Keywords:** emotional disorder, diagnosis, machine learning, eye movement, sports watching

## Abstract

**Introduction:**

Digital technologies have significantly advanced the detection of emotional disorders (EmD) in clinical settings. However, their adoption for long-term monitoring remains limited due to reliance on fixed testing formats and active user participation. This study introduces a novel approach utilizing common ball game videos–table tennis–to implicitly capture eye movement trajectories and identify EmD through natural viewing behavior.

**Methods:**

An eye movement data collection system was developed using VR glasses to display sports videos while recording participants' eye movements. Based on prior research and collected data, four primary eye movement behaviors were identified, along with 14 associated features. Statistical significance was assessed using t-tests and U-tests, and machine learning models were employed for classification (SVM for single-feature analysis and a decision tree for significant features) with k-fold validation. The reliability of the proposed paradigm and extracted features was evaluated using intraclass correlation coefficient (ICC) analysis.

**Results:**

Significance tests revealed 11 significant features in table tennis videos, encompassing exploration, fixation, and saccade behaviors, while only 3 features in tennis videos, which served as a supplemental stimulus, were salient in the re-testing. GazeEntropy emerged as the most predictive feature, achieving an accuracy of 0.88 with a significance *p*-value of 0.0002. A decision tree model trained on all significant features achieved 0.92 accuracy, 0.80 precision, and an AUC of 0.94. ICC analysis further confirmed the high reliability and significance of key features, including GazeEntropy and fixation metrics (average, maximum, and standard deviation).

**Discussion:**

This study highlights the potential of ball game video viewing as a natural and effective paradigm for EmD identification, particularly focusing on two key characteristics of EmD: curiosity exploration and psychomotor function. Additionally, participant preferences for video content significantly influenced diagnostic performance. We propose that future in-home, long-term monitoring of psychological conditions can leverage interactions with daily digital devices, integrating behavioral analysis seamlessly into everyday life.

## 1 Introduction

Neurological Disorders (ND) are the leading cause of disability-adjusted life years and the second leading cause of death globally, with over 3 billion people-about 43% of the world's population-affected reported in 2021 (1, 2). While medications combined with treatment strategies could effectively manage the severity of ND, alleviate symptoms, and enhance patient quality of life (3). However, as a major by-effect of prevailing medicines, emotional disorders (EmD) such as anxiety and depression are frequently observed as comorbid complications, particularly in people who manage multiple conditions and undergo polypharmacy (4). These psychological disturbances can significantly exacerbate preexisting conditions or trigger the development of new health issues, thereby complicating disease management (5–7). Addressing patients' emotional well-being is therefore critical, enabling clinicians to make informed adjustments to treatment plans. Timely recognition and effective management of these emotional complications enhance treatment outcomes and reduce the risk of further health deterioration. This highlights the integral role of mental health care within comprehensive disease management strategies.

Currently, the clinical diagnosis of EmD predominantly relies on psychological behavioral scales, such as the Hamilton Anxiety Scale (HAMA) (8), the Hamilton Depression Scale (HAMD) (9), and the Pittsburgh Sleep Quality Index (PSQI) (10). While widely used, the subject must undergo professional training before the assessment (11). Additionally, during the assessment, the subject must recall and describe situations from the past month, a process that may introduce bias (12). The interval between follow-up visits, ranging from 2 to 6 weeks, is unsuitable for frequent or prolonged monitoring of the medication's effects on the disease (13). Fortunately, advancements in innovative digital diagnostic methods offer promising solutions to these challenges. These approaches could enhance the consistency and efficiency of EmD assessment and enable more reliable long-term monitoring.

Eye movement (EM) signals have gained increasing attention as a valuable physiological data source in the study of EmD, in recent years. Compared to other biological signals, EM signals offer distinct advantages, including low-cost, robustness to noise, ease of acquisition, and independence from spatial constraints, making them a powerful tool for investigating individual visual behavior (14). These signals not only capture an individual's visual tracking patterns but also provide insights into visual preference characteristics through time-series data. Eye movement behaviors (EMBs), such as fixation, saccades, and smooth pursuit, are primarily regulated by the frontal eye field (15) and superior colliculus (16)–key brain regions whose functional abnormalities have been linked to EmD (17). Research has shown that individuals with EmD often exhibit atypical saccade patterns and impaired smooth pursuit abilities (18). Therefore, the design of experimental paradigms plays a critical role in EM research. By carefully structuring specific visual stimuli, experimental paradigms can effectively elicit emotional responses, enabling researchers to more precisely examine the relationship between EM patterns and EmD. Tasks such as pro-saccade and anti-saccade are commonly utilized for detecting EmD. Individuals with depression demonstrated an increased error rate in the anti-saccade task (19–21). Building on this, compared to the pro-saccade task, the anti-saccade task elicited similar results, including prolonged reaction times, which correlated with the severity of the disorder (22). These findings have also been observed in anxiety detection studies using the same task (23).

In contrast to intentionally induced EMs, recent studies have emphasized the importance of natural EMs–those detectable in everyday life. Significant differences have been identified between patients with major depression and healthy controls in smooth pursuit and free-viewing tasks, with variations observed in peak tracking speed, number of fixations, fixation duration, number of saccades, and saccade density (24). Additionally, anxiety and depression patients have been assessed using free-viewing tasks involving a matrix of 16 images from two distinct stimulus categories (25). Notably, fixation behaviors in free-viewing tasks were associated with greater prefrontal involvement compared to task-related settings (26). Some research suggests that emotion elicitation through photographs is relatively constrained, whereas emotional experiences evoked by aesthetic appreciation tend to be more profound (27). Major depression was assessed through the natural observation of oil paintings, achieving an accuracy rate of 79.88% by analyzing EM patterns, including heatmaps, trajectories, and statistical vectors (28). However, these tasks or the observation of static images often fail to sustain engagement, with even innovative approaches like observing oil paintings quickly inducing fatigue. For effective emotional monitoring, it is essential to develop a method that supports long-term use, ensures high user compliance, and aligns closely with everyday activities.

Task paradigms with dynamic visual stimulation have demonstrated effectiveness in research but are constrained by their rigid formats, limiting their practicality for long-term monitoring in daily life. While natural EM offers distinct differential characteristics and is better suited for everyday integration, static paradigms fail to generate sufficient cognitive arousal compared to task-based approaches. To address these shortcomings, this study investigates the use of round-based ball game videos, such as table tennis and tennis, as a novel experimental paradigm for EmD recognition. Ball game videos are common and widely accepted in daily life. They typically feature fixed camera angles with rapid ball movements across the video frame, mimicking the stimulation patterns of saccadic EMs. Despite the potential of dynamic video paradigms, only a limited number of studies have utilized them to explore ND, and none have specifically focused on EmD from our literature review. Therefore, this study aims to evaluate whether round-based ball game videos can serve as an effective dynamic, natural paradigm for eliciting EM patterns characteristic of EmD.

To explore the proposed paradigm, we developed an EM acquisition system for dynamic video playback using virtual reality (VR) glasses. VR devices offer immersive visual-spatial environments and are increasingly adopted for home use. Additionally, many VR systems now integrate eye-tracking technology to enhance user interaction, providing a reliable tool for capturing ocular movements and trajectories. It is worth noting that although VR devices were employed in this study to create a controlled and independent experimental environment, this paradigm is theoretically adaptable to any device equipped with eye-tracking capabilities. For the experiment, we curated 22 table tennis and tennis game videos from publicly available online sources and edited them to retain segments with fixed camera perspectives. Participants were exposed to 8 randomly selected videos, during which their EM data were recorded for subsequent analysis. The study extracted features characterizing four aspects of EM and conducted significance tests to evaluate their effectiveness in distinguishing individuals with EmD (IEmD) from healthy controls (HC). Furthermore, a Machine Learning (ML) model was implemented to validate the paradigm, achieving accurate EmD recognition rates and demonstrating the feasibility of this approach for EmD assessment.

## 2 Materials and methods

### 2.1 Participant information

A total of 11 IEmD and 12 HC participated in this study^1^. Patients were recruited from the Department of Neurology outpatient clinic at The First Hospital of Hebei Medical University and were diagnosed and screened by experienced clinicians. HC were recruited from the local community. All participants underwent assessments using the 24-item Hamilton Depression Rating Scale (HAMD-24), Hamilton Anxiety Scale (HAMA), and Pittsburgh Sleep Quality Index (PSQI). The demographic characteristics of the participants are presented in Table 1. The Ethics Committee of The First Hospital of Hebei Medical University reviewed and approved the study protocol. All participants provided written informed consent before their inclusion in the study.

**Table 1:** The demographic information table of participants.

	**EmD**	**HC**	**Sig**
**n (total = 25)**	**12**	**13**	**/**
**Demographics**			
Female #(%)	6 (50%)	10 (76.92%)	/
Age #(std)	47.33 (10.53)	48.31 (11.18)	/
Education yrs #(std)	12 (3.05)	9.08 (3.17)	< 0.05
**Clinical characteristic**			
HAMA avg (std)	14.42 (7.09)	4.31 (2.55)	< 0.001
HAMD avg (std)	18.08 (9.03)	4 (5.25)	< 0.001
PSQI avg (std)	13.67 (3.89)	8.38 (7.18)	< 0.05

The inclusion and exclusion criteria for this study were as follows:

- Inclusion criteria: (1) Participants aged 65 years or younger were all native speakers. (2) For the patient group, a confirmed diagnosis of depression or anxiety.
- Exclusion criteria: (1) Inability to complete the required examinations. (2) The presence of diseases causing bradykinesia, such as Parkinson's disease (PD), progressive supranuclear palsy (PSP), or multiple system atrophy (MSA). (3) Co-existing ocular conditions, such as glaucoma or cataracts, leading to blurred vision or visual field loss. (4) Disorders affecting ocular movement, such as oculomotor nerve damage or myasthenia gravis. (5) Recent use (within three months) of medications like amantadine, diazepam, or levodopa, accompanied by visual hallucinations. (6) Major systemic diseases, including tumors, cardiovascular or pulmonary conditions, or neurological disorders. (7) Diagnosed dementia conditions, including Parkinson's disease dementia (PDD), frontotemporal dementia (FTD), dementia with Lewy bodies (DLB), or normal pressure hydrocephalus (NPH).

### 2.2 Experimental

The study investigates whether EMB during natural video viewing can effectively detect EmD. Ball sports, commonly observed in daily life, often exhibit repetitive and cyclic motions, with the ball moving back and forth between players, as seen in table tennis. Given its popularity as a recreational activity, table tennis was chosen as a video paradigm to evoke EMs. In addition, to investigate whether the video content influenced EMs, we used tennis, an internationally popular ball sport, as a complementary stimulus. We opted for highlight reels of high-energy matches to maximize the natural and effective elicitation of EMs. These intense and competitive moments are engaging and effective in drawing attention. High-energy video clips were collected from official websites. Following screening based on criteria such as video clarity, duration, ball speed, and movement range, 22 video clips were finalized for use in the study, consisting of nine table tennis and 13 tennis.

**Figure 1:**
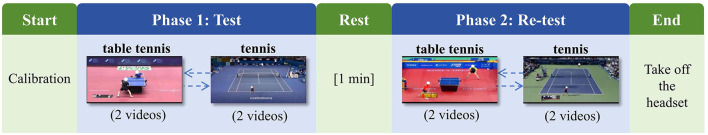
The experimental procedure for each participant.

The experiment was designed in two phases (as illustrated in Figure 1), with each phase comprising four video clips: two table tennis clips and two tennis clips. Since each video lasted an average of less than 20 seconds, the same type of videos was grouped to ensure participants could fully focus without being interrupted by frequent transitions. A Latin square design was employed to arrange the sequence of table tennis and tennis videos to eliminate order effects, resulting in two possible viewing orders.

The first phase served as the **Test**, while the second phase acted as the **Re-test**, primarily aimed at validating the hypotheses established during the test stage. In the re-test phase, the viewing order of the videos mirrored that of the test phase. Each video clip in both phases was randomly selected from a predefined collection, ensuring that the second video of the same content within a stage differed from the first. This design focused on the influence of video content on EMs rather than the specific content of individual video clips.

The experimenter managed all tasks throughout the experiment, ensuring participants remained fully immersed in the video experience. The experimental procedure was as follows: (1) The experimenter assisted participants in wearing the VR headset and instructed them to watch the videos as they would typically watch television. (2) Once participants were ready, the experimenter initiated the test by pressing the controller's trigger. The test began with an eye calibration process, after which the system automatically transitioned to the video playback screen. Upon completing the playback of four videos, participants were given a 1-minute break. (3) After the break, the experimenter triggered the second round of video playback using the controller. Once playback was complete, the experimenter removed the headset from the participants, marking the experiment's conclusion.

The visual environment for the experiment was created using virtual reality technology (detailed implementation in Section 2.5). All videos were presented in a completely dark environment on a 21.5-inch screen with a resolution of 1,280 × 720, positioned one meter from the participant's eyes. The experiment location was flexible but required a quiet setting to minimize distractions. Participants remained seated on a chair throughout the experiment to ensure comfort and stability.

### 2.3 Features definition

To effectively differentiate between HC and IEmD, researchers have investigated distinctions in daily behaviors between the two groups. However, describing human behavior poses significant challenges, often necessitating the extraction of behavioral characteristics to explore their relationship with disease status. These characteristics can be potential behavioral biomarkers for diagnosing and monitoring emotional disorders.

This study focuses on analyzing subjects' EMBs while watching videos. Initially, we review commonly used EM characteristics identified in previous research. Building on this foundation, we introduced several supplementary features to enhance behavioral analysis fitting the proposed novel paradigm. By categorizing and summarizing representative behavioral characteristics, we classified them into distinct behavioral types and analyzed their potential links to emotional disorders. Specifically, we identified four primary categories of behavior during video watching: **Exploration**, **Fixation**, **Saccade**, and **Attention**. Table 2 shows a detailed summarized table of features catalog.

**Table 2:** Features extracted based on different EMBs and their description.

**Features**	**Definition**	**Interpretation**
**Exploration**		
Fixation entropy	The entropy of gaze point during one video watching. Measures the randomness or unpredictability of gaze points during one video watching.	High entropy indicates dispersed and unpredictable gaze behavior, while low entropy suggests more restricted exploratory behavior.
KL-Divergence	The KL-divergence of eye distribution to ball distribution during one video watching.	Quantifying how eye-gaze distribution deviates from the ball distribution. Low divergence indicates goal-directed exploration with gaze aligned to task objectives, while high divergence suggests inefficiency or disengagement from task-relevant stimuli.
**Fixation**		
Number of fixation points	The number of distinct fixation behaviors during one video watching.	Fewer fixation behaviors reflect reduced exploration or interest in the video content.
Duratiuon_Avg	The average duration of individual fixation behaviors during one video watching.	Longer fixation durations and extended fixations may indicate difficulty disengaging or perseveration, while variability reflects rigid attention (low) or fluctuating focus (high).
Duratiuon_Max	The maximum duration of individual fixation behaviors during one video watching.	
Duratiuon_Std	The duration standard deviation of individual fixation behaviors during one video watching.	
**Saccade**		
Speed_Avg	The average speed of saccades during one video watching.	Reduced mean or maximum saccade speed may indicate psychomotor slowing or reduced engagement, while variability in speed reflects inconsistent focus (high variability) or uniformly restricted saccade (low variability).
Speed_Max	The maximum speed of saccades during one video watching.	
Speed_Std	Standard deviation of saccade speeds during one video watching.	
Amplitude_Avg	The average saccades moving Amplitude of the eyeball during one video watching.	Changes in saccade amplitude may indicate differences in exploratory behavior, with narrower or lower maximum amplitude reflecting reduced curiosity or engagement, while variability reveals fluctuating focus (high) or restricted saccade (low).
Amplitude_Max	The maximum saccades moving Amplitude of the eyeball during one video watching.	
Amplitude_Std	The standard deviation of saccades moving Amplitude of eyeball during one video watching.	
**Attention**		
Player1 attention ratio	The proportion of time spent looking at the upper player during the video.	Skewed attention ratios could indicate preferential focus or avoidance of specific visual regions, potentially tied to emotional processing or interest levels.
Player2 attention ratio	The proportion of time spent looking at the lower player during the video.	

#### 2.3.1 Exploration behavior

Exploration behavior refers to the overall watching patterns exhibited by video viewers throughout the video-watching process (29). This behavior captures how viewers engage with and visually interact with the video content.

Two key features are used to describe exploratory behavior:

***GazeEntropy:*** This feature reflects the randomness or dispersion of gaze points during video watching. Using *P*_*eye*_ denote as the distribution of gaze points during a whole video watching. The probability of a gaze point located on an independent point (*x*_*i*_) on the screen is *p*(*x*_*i*_). The entropy could be calculated as:


(1) $$\begin{array}{c} H\left(X\right)=-\overset{n}{\underset{i=1}{\sum }}p\left(x_{i}\right)\log p\left(x_{i}\right) \end{array}$$

Higher entropy indicates broad, exploratory behavior, while lower entropy suggests more focused or restricted exploration (30). This measure aligns with psychological theories of exploration, where individuals seek new information or broadly scan their environment (31).

***KL-divergence:*** This feature quantifies the difference between the viewer's EM distribution and the distribution of an expected object of interest, such as a moving ball in sports videos. The ball distribution *P*_*ball*_ works as the target distribution and the *P*_*eye*_ comparison distribution. For an independent point (*x*_*i*_) on the screen, the probability of gaze or ball appearing at this point is *p*_*e*_(*x*_*i*_) and *p*_*b*_(*x*_*i*_) respectively. So the KL-Divergence is denoted as:


(2) $$\begin{array}{c} D_{\text{KL}}\left(P_{eye}||P_{ball}\right)=\overset{n}{\underset{i=1}{\sum }}p_{e}\left(x_{i}\right)\log\frac{p_{e}\left(x_{i}\right)}{p_{b}\left(x_{i}\right)} \end{array}$$

The ball, as a salient visual point, often influences gaze behavior. A low KL-divergence indicates that the viewer's gaze closely follows the expected object, suggesting alignment with the anticipated pattern of attention.

#### 2.3.2 Fixation behavior

When processing visual information, individuals' gaze points often remain focused within a specific area for a while. This behavior, known as fixation, occurs when the eyes are relatively stable, concentrating on a particular point. Fixation behavior provides valuable insights into how individuals allocate attention, process information, and engage with videos (32, 33). By analyzing fixation patterns, researchers can infer cognitive strategies, emotional responses, and levels of task engagement. In this study, we focused on two key aspects of fixation behavior:

***Number of fixations:*** The total number of fixations during one video-watching indicates how visual attention is distributed over time (34). This feature provides insights into cognitive processing, task engagement, and emotional states.

***Fixation duration:*** This feature measures the time spent focusing on a specific area of interest. It is crucial for understanding the allocation of visual attention and cognitive resources (35). Fixation duration was assessed using the mean, maximum value, and standard deviation. The mean and maximum values reflect the efficiency of information processing in areas of interest, while the standard deviation indicates variability in focus depth and consistency of engagement.

#### 2.3.3 Saccade behavior

In ball game videos, where objects often move rapidly, viewers' areas of interest shift frequently, prompting significant saccadic activity throughout the watching process. Saccade behavior refers to the rapid EMs that occur between fixations (36). This behavior provides insights into changes in a viewer's area of interest during video watching. Two features-speed and amplitude-serve as indicators of EM dynamics and underlying cognitive functions.

***Saccade speed:*** Saccades are among the fastest movements produced by the human body, with speeds reaching up to 900°/*s* (37). During video watching, multiple saccadic movements are recorded. Key features derived from saccade speed include the mean, maximum value, and standard deviation. The mean and maximum values reflect physical and cognitive capabilities, while the variability (standard deviation) indicates the adaptability of eye behavior.

***Saccade amplitude:*** Amplitude refers to the distance between the start and end points of a saccade, measured in degrees of visual angle (38). Similar to speed, we analyze the mean, maximum value, and standard deviation of amplitude during video watching. The mean and maximum values reveal the extent of spatial exploration on the screen, while variability also serves as a measure of adaptability.

#### 2.3.4 Attention behavior

In the context of video watching, attention reflects how individuals allocate their focus to specific elements within the video (39). In this study, attention is defined as the proportion of time spent focusing on the players and balls, which are key visual elements that draw the viewer's attention in sports videos. To supplement the attention distribution, we calculate the time viewers spend fixating on the players.

***Player attention ratio:*** In ball games, players are typically positioned in the upper and lower halves of the screen with minimal overlap. Using an object recognition algorithm, we identify each player's bounding box. From the start of the video, the total time that the gaze points fall within the bounding box of a specific player is recorded. This value is then divided by the total video-watching time to compute the Player Attention Ratio, which represents the proportion of attention dedicated to a given player.

### 2.4 Features computation

Each feature was extracted from the original EM trajectory, ball movement trajectory, and player bounding box. For gaze entropy and KL-divergence, the first step is to convert both the EM and ball movement trajectories into two-dimensional probability distributions on the screen. For saccades and fixations, concentrated gaze points are identified from the EM time series, representing fixation behavior, while saccades are defined as the movements between these fixations. To calculate PlayerAttention, the number of fixation points within the player's bounding box is counted.

#### 2.4.1 Distribution

**Figure 2:**
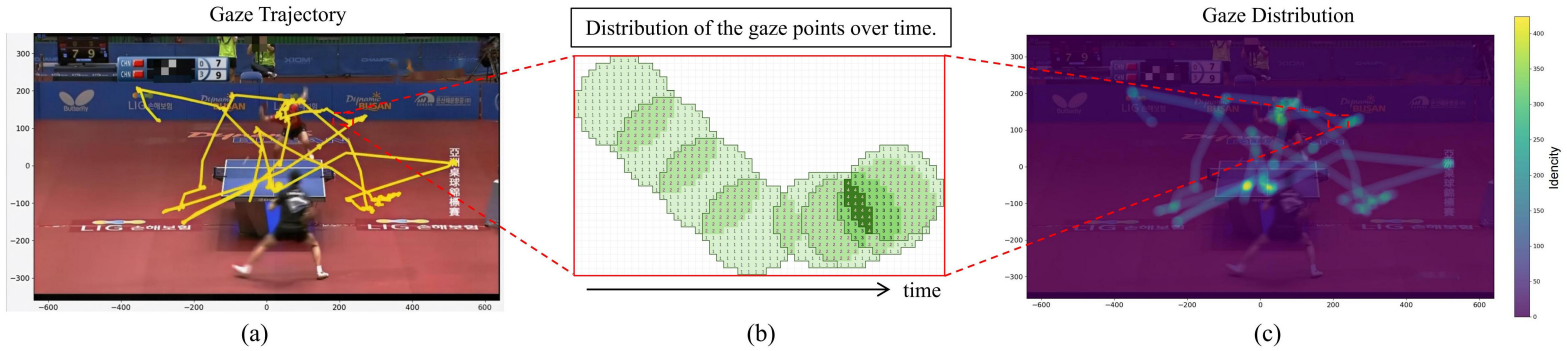
The process of calculating the distribution of moving gaze points on the screen. **(a)** shows the original eye movement trajectory. A circle with a fixed radius will move along the trajectory, and the area it passes through will accumulate 1, as shown in **(b)**. When moving to the final gaze point the final heat map is obtained, as shown in **(c)**.

To calculate the distributions of gaze and ball movement, each collected point is treated as the center of a circle with an adjustable radius. The circle moves along the trajectory of the target point, incrementing the value of the corresponding area on a screen-sized canvas by 1 for each position it covers. Once the entire trajectory is processed, the screen is represented as a two-dimensional distribution. The cumulative value at each point on the canvas is then normalized by dividing it by the total cumulative value across the entire canvas. This process yields the probability distribution of the target point on the screen (see Figure 2 for illustration).

Gaze entropy and KL-Divergence are computed using Formula 1 and Formula 2, respectively. It is important to note that the calculation of KL-Divergence involves division and logarithmic operations. These processes are sensitive to floating-point precision errors, which can cause problems when dealing with extremely small probabilities. For example, tiny numerical errors during summation or logarithmic operations may result in unexpected negative numbers due to rounding. To address these concerns, a minor constant (e.g., 1e-10) is added to the distribution to preclude division by zero or logarithmic operations on zero.

#### 2.4.2 Saccade and fxation

Eye-tracking data consists of a sequence of gaze points (*x, y, t*), where *x, y* are the screen coordinates of the gaze, and *t* is the corresponding timestamp. Clustering algorithms can effectively group these gaze points into clusters, each representing a fixation. A saccade is defined as the EM occurring between two consecutive fixations. The key features of a saccade, such as amplitude and speed, are derived from the spatial and temporal transitions between fixations.

**Figure 3:**
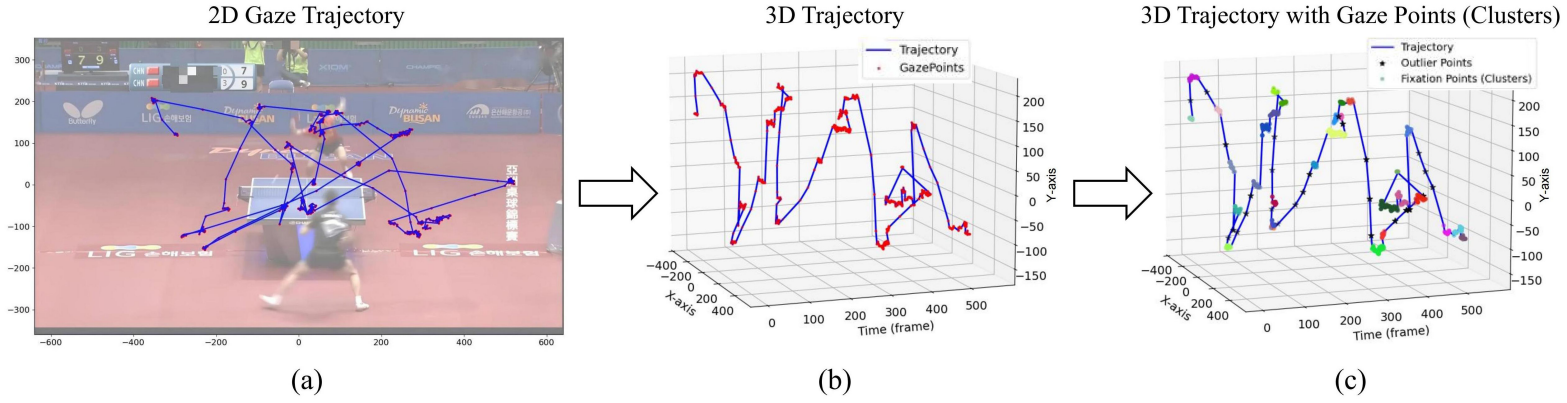
The process of clustering the fixation points using the raw gaze trajectory. From a 2D perspective, the eye movement trajectory exhibits overlapping gaze points over time, as illustrated in **(a)**. By introducing an additional time dimension, a 3D eye movement trajectory is generated, as depicted in **(b)**. Finally, clustering is applied to the 3D trajectory, resulting in distinct fixation points, as shown in **(c)**.

This study employs the Density-Based Spatial Clustering of Applications with Noise (DBSCAN) algorithm to group gaze points into fixations (40). By setting appropriate parameters for spatial distance (15 pixels, approximately equivalent to 1° of visual angle) and the minimum number of points (5 points), DBSCAN clusters closely spaced gaze points as fixations. Unlike algorithms that require a predefined number of clusters, DBSCAN identifies clusters based on point density, making it particularly suitable for scenarios where the number of fixations varies. This density-based approach also effectively handles the irregular and variable distribution of gaze points. This process is illustrate in Figure 3.

**Figure 4:**
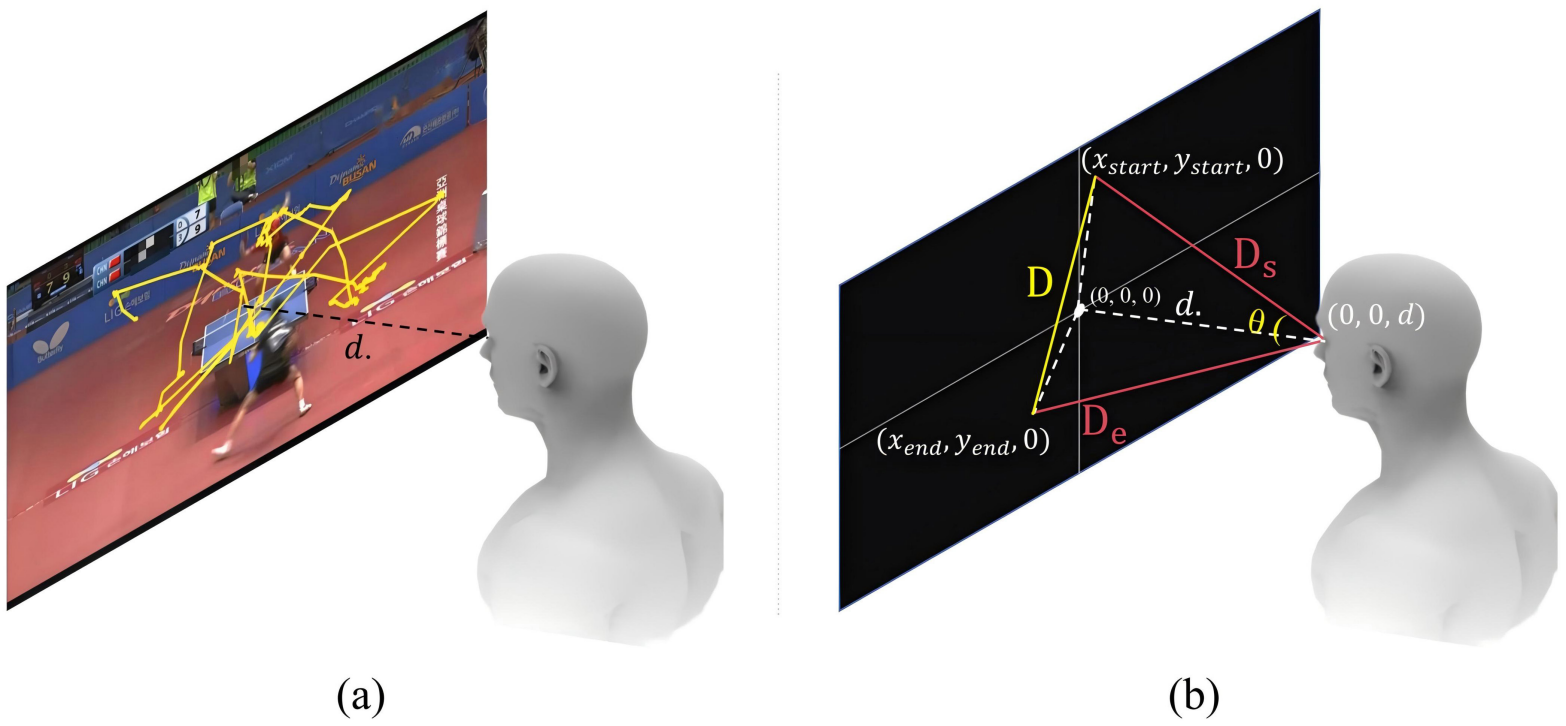
**(a)** Illustrates the gaze movement trajectory during video viewing, with a fixed eye-to-screen distance of 1 m (denoted as *d*). **(b)** Presents the method for calculating the gaze movement angle. The eye's flat (neutral) position is defined at the coordinate origin, corresponding to the center of the screen. Given the known start and end points of the gaze, the gaze movement distance (*D*) can be computed. The distances *D*_*s*_ and *D*_*e*_ are derived using the Pythagorean Theorem. With all three sides of the triangle determined, the gaze movement angle (θ) can be calculated using the Law of Cosines.

Since the collected data consists of pixel coordinates within a VR environment, it is necessary to convert these coordinates into real-world EM angles. This process begins by transforming the pixel coordinates into real-world distances, utilizing the scale parameter specified in the VR settings. Given the fixed one-meter distance between the video canvas and the viewer's eyes in the VR setup, the distances from the viewer's eyes to the start and end points of a saccade (denoted as *D*_*s*_ and *D*_*e*_, respectively) can be determined using the Pythagorean theorem. Using these distances and the Euclidean distance between the start and end points (*D*), the saccade amplitude, represented as the angle of EM, can then be calculated using the law of cosine (see Figure 4):


(3) $$\begin{array}{c} \cos\left(\theta \right)=\frac{D_{s}^{2}+D_{e}^{2}-D^{2}}{2D_{s}D_{e}} \end{array}$$

#### 2.4.3 Player attention

To calculate the proportion of time a viewer's gaze falls within an athlete's range, it is first necessary to identify the bounding box of the active athlete in the video. This study uses Deep-EIoU (41) to extract the bounding box for each athlete in every video frame, recording the coordinates of the upper-left and lower-right corners. Next, the algorithm identifies and counts the gaze points that overlap with the bounding box in each frame. Given the fixed sampling rate of eye-tracking data, the number of gaze points within the bounding boxes of the two athletes is multiplied by the time interval (20 ms) to calculate the total gaze duration for each player. Finally, the total gaze time for each player is divided by the overall eye-tracking duration to derive the attention ratio.

### 2.5 Implementation

We utilized Unity3D (Version 2021.3.26)^2^ to develop a VR-based video playback application, which was integrated into an intelligent eye movement analysis and evaluation system known as “EyeKnow.” This system is a medical device approved by the National Medical Products Administration and supports eye-tracking data acquisition at frequencies of up to 90 Hz (50 Hz in this study), with integrated real-time eye-tracking functionality.

Data processing was conducted primarily using Python (version 3.11),^3^ with several third-party libraries employed for specific tasks. The DBSCAN algorithm for fixation identification was implemented using Scikit-learn,^4^ while Deep-EIoU^5^ was used to extract player bounding boxes.

To validate the proposed features, we employed a parameter significance testing approach. The Shapiro-Wilk test was used to assess the normality of these features. Features following a normal distribution were evaluated using a t-test, while those deviating from normality were analyzed with a U-test. These statistical methods were implemented using the SciPy library.^6^ Additionally, we developed machine learning models to classify individuals into different groups based on the extracted features. For single-feature classification, a Support Vector Machine (SVM) with a linear kernel was employed. This excels in portraying the distinction between the two types directly through a linear method, offering simplicity, efficiency, and interpretability. Incorporating all significant features, a decision tree model with a maximum depth of 5 was used. Both models were implemented using Scikit-learn.

## 3 Results

This section presents the analysis of data collected during the experiments. The primary focus is on evaluating the significance of the extracted features to investigate behavioral differences between IEmD and HC during video watching. To further validate the effectiveness of the proposed paradigm, we also examine the performance of ML models. These models demonstrate potential for clinical decision support and automated in-home alert systems, enhancing the practical applicability of this paradigm.

### 3.1 Features significance

**Figure 5:**
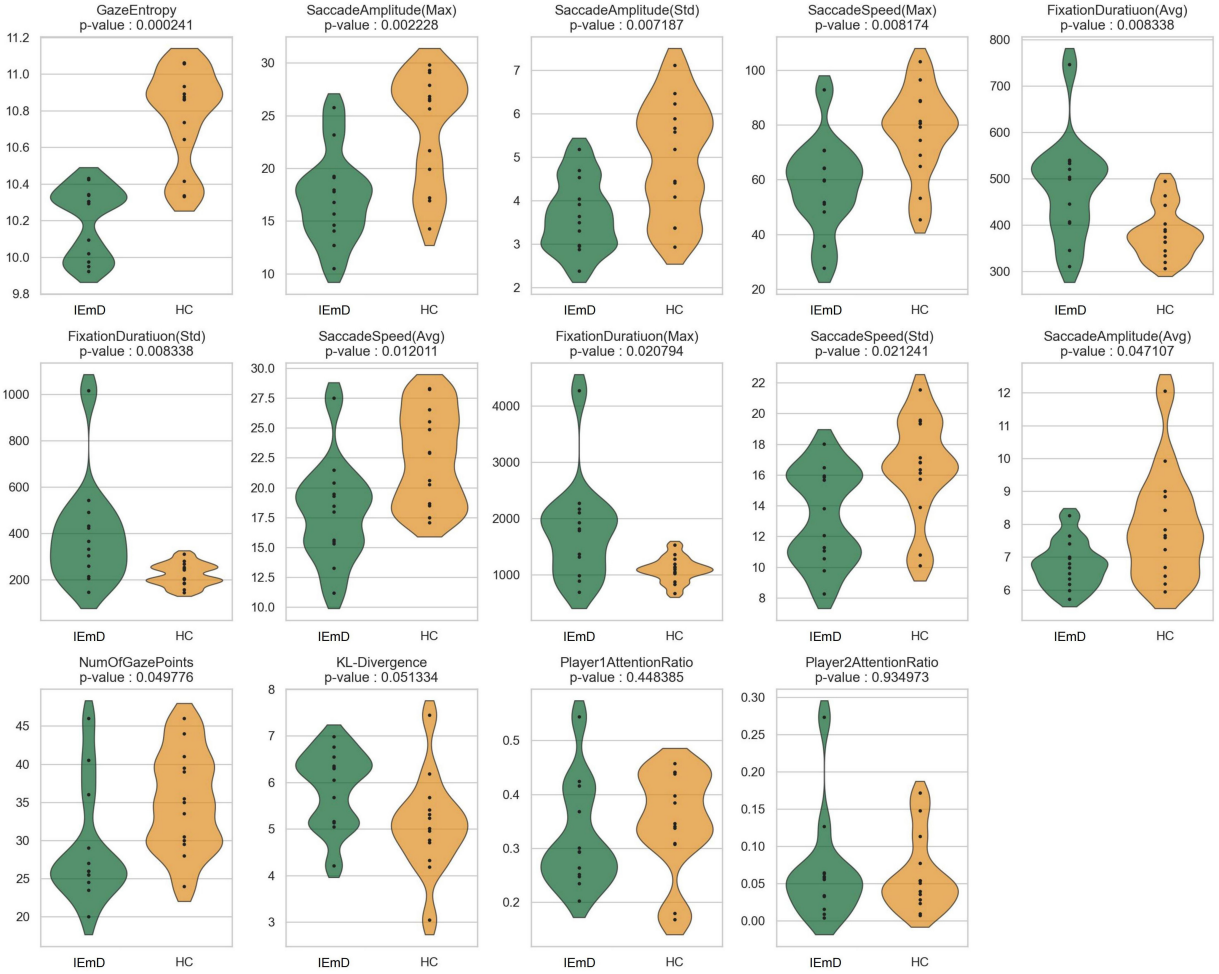
The violin plot of features of different participant groups during table tennis video watching (test phase).

The significance test could determine whether the observed differences or relationships in the data are statistically meaningful or merely attributable to chance. These tests provide a quantitative basis for validating hypotheses, reducing uncertainty, and guiding data-driven conclusions in research. Generally, if the p-value in the significance test is less than 0.05, the feature is considered significant, and the smaller the value, the stronger the significance. The results of the significance test are shown in Table 3, and the distributions of individual features are shown in Figure 5.

**Table 3:** The evaluation and re-evaluation results of all EMB features for the EmD across the two video types.

**Features**	**Test**				**Re-test**			
	**Table tennis**		**Tennis**		**Table tennis**		**Tennis**	
	**Acc**	**p_value**	**Acc**	**p_value**	**Acc**	**p_value**	**Acc**	**p_value**
**Exploration**								
GazeEntropy	0.88	**0.0002**	0.36	0.5679	0.64	**0.0013**	0.64	**0.0307**
KL-Divergence	0.68	0.0513	0.40	0.7373	0.68	**0.0266**	0.64	0.2419
**Fixation**								
Number of Fixation Points	0.68	**0.0498**	0.36	0.9850	0.76	**0.0018**	0.64	0.0633
Duratiuon_Avg	0.72	**0.0083**	0.32	0.7900	0.68	**0.0025**	0.60	0.3992
Duratiuon_Max	0.76	**0.0208**	0.56	0.2107	0.80	**0.0046**	0.36	0.8917
Duratiuon_Std	0.76	**0.0083**	0.48	0.3013	0.80	**0.0018**	0.40	0.2648
**Saccade**								
Speed_Avg	0.64	**0.0120**	0.36	0.8933	0.64	**0.0064**	0.48	0.1996
Speed_Max	0.76	**0.0082**	0.32	0.7238	0.68	**0.0244**	0.64	0.2621
Speed_Std	0.64	**0.0212**	0.40	0.8309	0.64	**0.0382**	0.68	0.1573
Amplitude_Mean	0.64	**0.0471**	0.40	0.8311	0.56	**0.0412**	0.56	0.2235
Amplitude_Max	0.72	**0.0022**	0.52	0.6521	0.60	**0.0128**	0.68	**0.0083**
Amplitude_Std	0.72	**0.0072**	0.60	0.3257	0.68	0.0535	0.64	**0.0414**
**Attention**								
Player1 Attention Ratio	0.56	0.4484	0.36	0.7606	0.28	0.8995	0.48	0.7217
Player2 Attention Ratio	0.44	0.9350	0.36	0.6238	0.52	0.1632	0.44	0.7237

Bolded content indicates that the *p*-value is significant, i.e., less than 0.05.

Overall, during the table tennis video watching, most features demonstrated strong statistical significance, with 11 features proving effective in both the test and re-test phases. The subsequent content provides a detailed analysis of each feature type.

#### 3.1.1 Exploration

The Exploration behavior category includes two features: GazeEntropy and KL-Divergence.

**GazeEntropy** exhibited the highest significance, with a p-value of 0.0002 in the test phase and 0.0013 in the re-test phase. The GazeEntropy values for HC were higher than IEmD. This finding suggests that HC exhibited more active exploration behavior compared to IEmD when watching these videos.

**KL-divergence** was significant only during the re-test phase, with a generally similar distribution between HC and IEmD. However, when KL-Divergence reached significance (see Supplementary Figure 1), HC participants exhibited lower values than those in the IEmD group. Comparable KL-Divergence values between groups suggest that both directed their attention toward similar regions of interest, primarily aligned with the ball's movement in the video. When significant, the lower KL-Divergence values observed in HC participants indicate a more focused and consistent attention toward the ball's trajectory.

Regarding exploratory behavior, HC were more active, exploratory, and conformed to the movement of the ball, whereas the opposite was true for IEmD.

#### 3.1.2 Fixation

Fixation behavior consists of the Number and the Duration of Fixations.

**Number of fixation points** of HC were larger than that of IEmD, indicating that HC were more engaged.

**Fixation duration (Avg. and Max)** of HC had shorter than that of IEmD, indicating a greater involvement with table tennis' rapid pace. In contrast, the longer average fixation lengths seen in IEmD may represent issues adjusting to the game's fast-paced nature (related to cognitive function) or a loss of interest in the material (associated with depression). **Fixation duration (std)** of HC had a reduced standard deviation, indicating more uniform fixation behaviors during gameplay.

These findings of the fixation behavior suggest that HC are more likely to follow the rhythm of the game, indicating higher engagement and alignment with the video content across both video types.

#### 3.1.3 Saccade

Saccade behavior is characterized primarily by two features: speed and amplitude.

The results revealed differences in **Saccade speed (Avg. and Max)** between the groups, with higher values observed in HC compared to IEmD. The reduced saccade speed in individuals with IEmD may suggest decreased motor system efficiency, potentially indicating psychomotor slowing. Additionally, **Saccade speed (Std.)** was greater in HC, suggesting higher engagement and a greater ability to adapt to rapid changes in game pacing. This increased variability in saccadic movements further reflects the stronger psychomotor capabilities of HC in tracking the dynamic nature of table tennis.

**Saccade amplitude (Avg. and Max)** also showed different distribution. HC exhibiting greater amplitudes than those in IEmD. This finding suggests that HC engaged in a broader visual exploration of the screen, whereas the more restricted gaze exploration observed in IEmD may reflect diminished curiosity, a characteristic often associated with EmD. Similarly, during the test phase, **Saccade amplitude (Std)** with HC displayed greater variability. This increased variability may be attributed to a combination of heightened exploratory curiosity and active observation. A stronger curiosity enables participants to scan a larger area of the video, while more engagement results in more diverse EMs as they track dynamic changes in the video.

#### 3.1.4 Attention

Attention behavior was assessed based on the proportion of time participants focused on the athlete's area while watching the game. The experimental results indicated no significant differences between groups during the video-watching or test phases. However, the data showed that the **Player1AttentionRatio** was consistently higher, suggesting that both HC and IEmD participants directed more attention toward the athletes at the top of the screen. Given the simplicity of the attention measurement method employed in this study, further differentiation of attentional behaviors could not be derived from this observation.

### 3.2 Recognition result

The SVM with a linear kernel complements the significance tests by assessing the predictive power of each feature. The accuracy of the SVM offers additional evidence of the features' ability to distinguish between classes, highlighting their practical utility beyond statistical validation. To further assess the discriminative capacity of this new paradigm, we constructed a decision tree with a maximum depth of 5 using all significant features to develop a recognition model. In the analysis, K-Fold validation (*k* = 5) was used. K-fold cross-validation is a resampling method used to evaluate model performance by dividing the dataset into k equally sized folds, testing on one fold, and training on the remains. This approach ensures that every data is used for both training and testing, reducing overfitting and providing a more reliable estimate of model generalization. Given the lack of a standardized accuracy metric and the study's focus on binary classification, we propose the following accuracy benchmarks: values below 0.5 are considered poor, 0.5–0.7 moderately good, 0.7–0.9 good, and above 0.9 very good.

#### 3.2.1 Single feature classification

In table tennis watching, no single feature achieved a “very good” accuracy level, though most features reached the “good” level. During the test phase, seven features demonstrated good recognition performance, with GazeEntropy achieving the highest accuracy of 0.88. The remaining six features were categorized as “moderately good,” with only one classified as “poor.” In the re-test phase, overall recognition performance declined, with three features classified as “good,” ten as “moderately good,” and one as “poor.”

Overall, the results of single-feature recognition for IEmD largely align with the findings of the significance tests, as significant features consistently provided better recognition performance. The highest recognition accuracy, 0.88, further validates GazeEntropy as an effective biomarker in video watching differentiating IEmD. Between the two paradigms, table tennis outperformed tennis in recognition accuracy–a difference explored in subsequent discussions.

#### 3.2.2 Overall recognition capacity

In classification tasks, several key metrics are used to evaluate model performance. Accuracy (Acc.) measures the proportion of correctly classified instances over the total, offering an overall correctness. Precision (Pre.) measures the proportion of true positives among predicted positives, while Recall quantifies the proportion of true positives correctly identified by the model. The F1 score, a harmonic mean of precision and recall, balances these two metrics. Lastly, the Area Under the Curve (AUC) of the Receiver Operating Characteristic (ROC) curve assesses the model's ability to distinguish between classes across various threshold settings, offering a robust measure of discriminative power. The results of evaluating the model are listed in Table 4.

**Table 4:** The metrics of the Decision Tree recognition model training on significant features (Tennis video watching in the test phase used all features).

		**Acc**.	**Pre**.	**Recall**	**F1**	**AUC**
Table tennis	Test	**0.92**	0.80	**0.69**	**0.74**	**0.94**
	Re-test	0.80	0.73	0.63	0.65	0.76
Tennis	Test	0.60	0.42	0.60	0.47	0.70
	Re-test	0.76	**0.83**	0.67	0.71	0.77

Bolded content indicates the best result in each assessment metric.

As for the table tennis watching, the test phase demonstrates strong performance across all metrics, with high accuracy (0.92), F1-score (0.74), and AUC (0.94). This indicates that the model can distinguish between classes effectively during this phase. However, recall (0.69) is lower than precision (0.80), suggesting that while positive predictions are mostly correct, some true positives are being missed. The re-test phase shows a drop in performance across all metrics compared to the test phase. Accuracy drops to 0.80, and AUC falls to 0.76, reflecting reduced discriminative ability during re-testing. This may point to variability in model performance over time or participant inconsistency in behavior.

To gain deeper insight into the factors shaping the model's classification performance, we used the Gini importance to determine which features play the most important role in distinguishing between groups. This metric quantifies the extent to which features influence the model's decision-making process, by calculating the (normalized) total reduction of the criterion brought by that feature. The analysis revealed that **GazeEntropy**, **SaccadeSpeed (Max)** and **GazeDuration (Std)** were the top three influential features, with the values of 0.3915, 0.1704, and 0.1693, respectively. Notably, these features also exhibited significant differences in our earlier feature analyses, reinforcing the relevance of the extracted features in classification.

### 3.3 Video content influences

This section extends the analysis of gaze behavior in tennis video to examine the impact of video content influences on visual behavior. It was discovered that the number of features with significant differences in the tennis video was substantially lower than the procedure for the table tennis video, and no features with significant differences were extracted during the test phase of the tennis video. Tennis is less familiar to Chinese people than table tennis, which could explain why. The Section 4 will go into greater detail and explain everything.

#### 3.3.1 Feature significance

**Figure 6:**
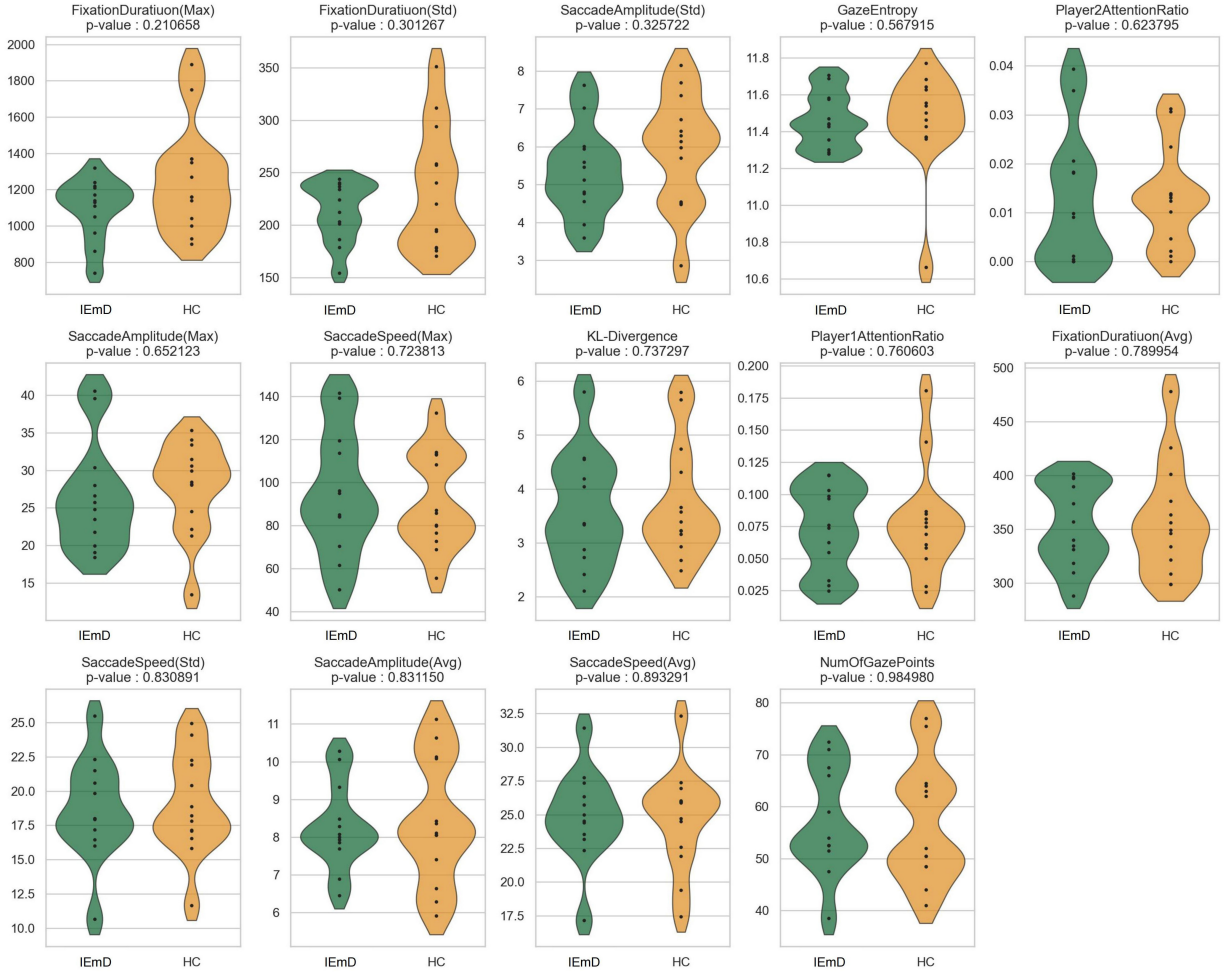
The violin plot of features of different participant groups during tennis video watching (test phase).

Watching the tennis videos, GazeEntropy and Saccade Amplitude exhibited significant differences in the re-test phase, while no features differed significantly in the test phase, as illustrated in Figure 6 and Supplementary Figure 2.

**GazeEntropy** showed statistical significance (*p* = 0.0083), with HC having higher entropy values than IEmD in the re-test phase. This conclusion is congruent with the results obtained in the table tennis video, implying that HC participated in more active exploratory behavior than their IEmD counterparts while watching the videos.

**Saccade amplitude (Max)** and **Saccade amplitude (Std)** showed differences in the distribution of the two populations at *p* = 0.0307 and *p* = 0.0438, respectively. The significance of Saccade Amplitude (Std) is in line with the results obtained for the table tennis video, where the HC showed a higher degree of variability in the re-testing phase of the tennis video viewing compared to that of the IEmD.

#### 3.3.2 Recognition result

Single Feature Classification: In the tennis matches, only two features reached the “moderately good” level during the test phase, while the remaining features showed accuracies below 0.5. However, recognition accuracy improved in the re-test phase, with nine features achieving “moderately good” levels and the highest accuracy reaching 0.68.

Overall Recognition Capacity: The tennis watching shows weaker performance in the test phase, with accuracy (0.60), F1-score (0.47), and AUC (0.70) being relatively low. Precision (0.42) is notably poor compared to recall (0.60), indicating a tendency to produce false positives. The re-test phase shows improved performance, with accuracy increasing to 0.76 and AUC reaching 0.77. Precision (0.83) surpasses recall (0.67), suggesting a stronger ability to classify positive predictions with fewer false positives during re-testing correctly. The model performs significantly better on the table tennis task in the test phase, indicating that the task's features or dynamics may align better with the model's discriminative capabilities. Additionally, we employed Gini importance to quantify the contribution of each feature to the classification. The analysis revealed that **GazeEntropy**, **GazeDuration (Max)**, and **Player2AttentionRatio** were the most influential features in the tennis video.

### 3.4 Test and re-test

The Intraclass Correlation Coefficient (ICC) is a statistical measure used to assess the reliability or consistency of measurements across different circumstances. ICC values range from 0 to 1, with higher values indicating stronger reliability; commonly, ICCs above 0.75 are considered excellent, while values between 0.5 and 0.75 indicate moderate reliability, and those below 0.5 suggest poor reliability. In this study, ICC3k was used in the assessment. ICC3k is a specific type of ICC used to assess the consistency or agreement of measurements when the same raters (the rater means the feature-extracting method in this study) are applied to all subjects and the focus is on reliability across multiple measurements.

The results of ICC analysis are listed in Table 5. In table tennis watching, the ICC results suggest that GazeEntropy and KL-Divergence are the most reliable features for distinguishing gaze behavior, as evidenced by their high ICC values and narrow confidence intervals. In contrast, features such as attention ratio for Player2AttentionRatio and Saccade Speed (Std) demonstrated lower reliability, indicating potential variability or measurement inconsistencies.

**Table 5:** Results of ICC analysis of individual features across different game watching.

**Features**	**Table tennis**			**Tennis**		
	**ICC**	**p_value**	**CI 95%**	**ICC**	**p_value**	**CI 95%**
**Exploration**						
Fixation Entropy	0.89	0.0000	[0.75 0.95]	0.41	0.1003	[-0.33 0.74]
KL-Divergence	0.79	0.0001	[0.53 0.91]	0.54	0.0318	[-0.05 0.8 ]
**Fixation**						
Number of Fixation Points	0.53	0.0335	[-0.06 0.79]	0.26	0.2368	[-0.69 0.67]
Duratiuon_Avg	0.69	0.0031	[0.29 0.86]	0.79	0.0001	[0.53 0.91]
Duratiuon_Max	0.65	0.0059	[0.21 0.85]	0.55	0.0283	[-0.02 0.8 ]
Duratiuon_Std	0.69	0.0029	[0.3 0.86]	0.61	0.0129	[0.11 0.83]
**Saccade**						
Speed_Avg	0.79	0.0002	[0.52 0.91]	0.43	0.0851	[-0.28 0.75]
Speed_Max	0.52	0.0400	[-0.09 0.79]	0.35	0.1486	[-0.47 0.71]
Speed_Std	0.40	0.1096	[-0.36 0.74]	0.35	0.1476	[-0.47 0.71]
Amplitude_Mean	0.65	0.0061	[0.21 0.85]	0.43	0.0855	[-0.29 0.75]
Amplitude_Max	0.62	0.0107	[0.14 0.83]	0.17	0.3277	[-0.89 0.63]
Amplitude_Std	0.62	0.0099	[0.15 0.83]	0.46	0.0684	[-0.22 0.76]
**Attention**						
Player1 Attention Ratio	0.67	0.0040	[0.26 0.86]	0.24	0.2556	[-0.73 0.66]
Player2 Attention Ratio	0.16	0.3401	[-0.91 0.63]	0.66	0.0057	[0.22 0.85]

In tennis watching, KL-Divergence, Player2AttentionRatio, and GazeDuratiuon (Avg., Max, Std.) are reliable features in the ICC analysis. The remaining features seem to be unreliable. In general, the features describing the two behaviors of Exploration and Fixation are reliable in two different video watching.

The model performs significantly better on the table tennis task in the test phase, indicating that the task's features or dynamics may align better with the model's discriminative capabilities. These results suggest task type and participant engagement significantly impact model performance over repeated sessions.

## 4 Discussion

This study leverages the rapid movement of the ball in turn-based sports, such as table tennis and tennis, as a visual stimulus to induce the gaze movement of participants. By analyzing 4 kinds of behaviors in video watching, we extracted effective features from the significance tests and facilitated the detection of EmD ML models. Experiments involving 25 participants identified 11 significant features in table tennis video watching, encompassing three behavioral categories. A decision tree model trained on all significant features achieved an accuracy of 0.92. Additionally, ICC analysis further confirmed that four features–GazeEntropy and Fixation (Average, Maximum, and Standard Deviation)-exhibited both high significance and reliability. These findings demonstrate the potential of behavioral analysis during video watching of ball games as an effective paradigm for identifying IEmD. This work aims to advance the application of EMBs in everyday contexts, ultimately supporting long-term, in-home EmD monitoring through advanced computational technologies. There are a few insights that could further enrich the future of this realm of research.

### 4.1 Key characters of EmD revealed in ball game watching: curiosity exploration and psychomotor

Our experimental findings indicate that key features for detecting EmD are closely linked to two primary characteristics of EmD: reduced curiosity exploration and psychomotor slowing. Specifically, GazeEntropy, Saccade Amplitude, and Gaze Duration are associated with curiosity exploration, as EmD often leads to decreased motivation and engagement with the environment. Meanwhile, Saccade Speed is related to psychomotor function, with EmD frequently causing psychomotor slowing, reflected in slower saccadic movements as an indicator of impaired motor and cognitive processing. Building on these insights, we can design more naturalistic paradigms for EmD detection. For instance, a video-based task requiring participants to explore a scene for specific objects could combine exploratory behavior and saccadic efficiency, thus assessing curiosity and psychomotor functioning. Additionally, adaptive paradigms that increase in complexity over time could challenge participants to balance curiosity-driven exploration with efficient psychomotor responses. Furthermore, integrating eye-tracking data with physiological measures such as heart rate variability (HRV) (42) or skin conductance (43) could enhance detection by linking psychomotor and curiosity patterns with emotional arousal or stress levels. These multi-modal approaches would offer a more comprehensive understanding of EmD and improve detection capabilities.

### 4.2 The effects of participants' preference of video contents to the diagnosis performance

The experiment revealed that features' significance, recognition accuracy, and reliability were notably higher for table tennis videos than tennis videos. Discussions with participants indicated that this discrepancy might stem from their familiarity with the sports. Several participants reported being unfamiliar with tennis, which hindered their ability to focus and engage with the content. In contrast, their familiarity with the rules and gaming strategy in table tennis allowed for greater concentration during watching. These findings suggest that using video content aligned with participants' preferences or familiarity could yield more accurate diagnostic information. However, unfamiliar content is not without diagnostic value. In the short test and re-test process, some features became significant during the re-test phase, indicating that the ability to adapt and learn from novel video content could itself serve as a potential biomarker for EmD. Further research is needed to explore this hypothesis. In practical applications, exposing participants to a variety of video content may enhance the robustness and usability of this method, accommodating both familiar and unfamiliar stimuli to capture a broader range of diagnostic indicators.

### 4.3 The future of in-home long-term monitor for psychology disease: daily digital devices interaction

This study, alongside recent research, highlights a growing shift from traditional digital medical paradigms to naturalistic paradigms in various fields, not only EmD but also other neurological conditions. While electronic traditional paradigms have achieved significant success in clinical testing-offering quick application, objective indicators, and ease of use during consultations-they face challenges when applied to daily monitoring due to their rigid and often unengaging nature. In response, researchers have explored paradigms based on common daily behaviors, such as reading tasks (44) or oil painting viewing (28). However, these still require active participation, limiting their practicality for passive, everyday use. A promising alternative is leveraging interactions with electronic devices commonly used in daily life. For example, prior studies have demonstrated the potential of mobile phone gestures for detecting Parkinson's disease (45). This study adopts a similar approach, using EM trajectories during video watching for EmD detection. Furthermore, this method can be extended to a variety of devices, including smartphones and smart TVs, enabling seamless, unobtrusive monitoring. By integrating disease detection into routine interactions, this approach minimizes disruption to daily life and enhances the feasibility of long-term monitoring.

### 4.4 Further improvements

Given that video content preferences significantly influence the relevance of extracted features, future research could incorporate content preference as an additional factor to enhance the performance of the proposed method. Integrating this factor into passive acquisition techniques may improve classification accuracy and provide a more effective approach for monitoring emotional states. For instance, in long-term monitoring, grouping preference data for detection and prioritizing highly preferred video content could yield more stable monitoring outcomes. Additionally, directly incorporating preference scores as features in machine learning models may enhance predictive performance by allowing the model to assign different weights to features based on user preferences, thereby improving overall accuracy.

Watching ball sports videos in a non-intrusive, non-stimulating, and engaging manner can be seamlessly integrated into daily life, enabling long-term passive monitoring of an individual's emotional state. Furthermore, this method could incorporate with other established methods for more reliable performance. For example, electroencephalography (EEG) offers high temporal resolution by capturing the brain's electrical activity in EmD recognition (46); functional MRI (fMRI) provides high spatial resolution by mapping functional brain activity (47). These modalities could be integrated during the video watching to allow for a more comprehensive representation of dynamic brain activity by combining complementary temporal and spatial information.

This study primarily assessed the feasibility of the proposed paradigm by evaluating accuracy as a key metric. The decision tree model currently in use meets this criterion. However, for broader applications in clinical or daily life settings, the paradigm may encounter increased variability due to larger and more diverse datasets. To enhance adaptability, future implementations could incorporate more advanced models, such as ensemble methods or deep learning approaches, to achieve a more balanced recognition performance. To explore this potential, we evaluated three ensemble models—Adaboost, Random Forest, and Gradient Boosting Decision Tree–as summarized in Table 6. The results indicate that all three models achieved accuracy comparable to that of the basic decision tree. Notably, the Adaboost model exhibited a more balanced trade-off between precision and recall while maintaining high overall accuracy, underscoring its effectiveness in improving classification performance.

**Table 6:** Effectiveness of three ensemble models and basic decision tree in detecting emotional disorders for the table tennis video method.

	**Acc**.	**Pre**.	**Recall**	**F1**	**AUC**
Decision tree	0.92	0.80	0.69	0.74	0.94
Adaboost	0.88	0.90	0.88	0.86	0.92
Random Forest	0.80	0.90	0.82	0.80	0.84
Gradient Boosting Decision Tree	0.88	1.00	0.82	0.87	0.91

### 4.5 Limitations

While this study has produced promising results, several limitations should be acknowledged. First, the sample size was relatively small and predominantly focused on cases of depression and anxiety. Although these are common forms of EmD, other EmD types were not included, which limits the study's generalizability. Additionally, the small participant group may not adequately represent broader populations. Nevertheless, the proposed paradigm and feature extraction method demonstrated effectiveness and consistency with previous findings. Given the method's practicality, it holds significant potential for large-scale research and applications in the future. From a methodological perspective, this study utilized only two types of ball game videos, tennis and table tennis. The applicability of these findings to other video genres remains uncertain and warrants further investigation. Lastly, the use of VR glasses in the experiment provided a controlled environment, reducing external distractions. However, when applying this method to more commonly used devices, such as smartphones or computers, users are likely to encounter environmental interruptions. To ensure the method's robustness across various platforms, further optimization will be necessary.

## Data Availability

A de-identified version of the dataset generated from the experiments, including demographic information, clinical scale scores, and the number of data points in the dataset, is shown in the footnote. A de-identified version of the dataset generated from the experiments, including demographic information, clinical scale scores, and raw eye-tracking data, has been curated and is available upon request under a controlled access protocol. Interested researchers may obtain access by contacting the first author via email qiangwei191@mails.ucas.edu.cn.

## Footnotes

^1^A de-identified version of the dataset generated from the experiments, including demographic information, clinical scale scores, and raw eye-tracking data, has been prepared under a controlled access protocol. Researchers interested in accessing the data may request it by contacting the first or corresponding author via email.

^2^ https://unity.com/releases/editor/whats-new/2021.3.26

^3^ https://www.python.org/downloads/release/python-31111/

^4^ https://scikit-learn.org/1.4/index.html

^5^ https://github.com/hsiangwei0903/Deep-EIoU

^6^ https://docs.scipy.org/doc/scipy/
