# Supplementary figures and images for: Detecting emotional disorder with eye movement features in sports watching
Source: Front Neurol. 2025 Apr 29;16:1562785. doi: 10.3389/fneur.2025.1562785 (PMC12069065; doi:10.3389/fneur.2025.1562785)

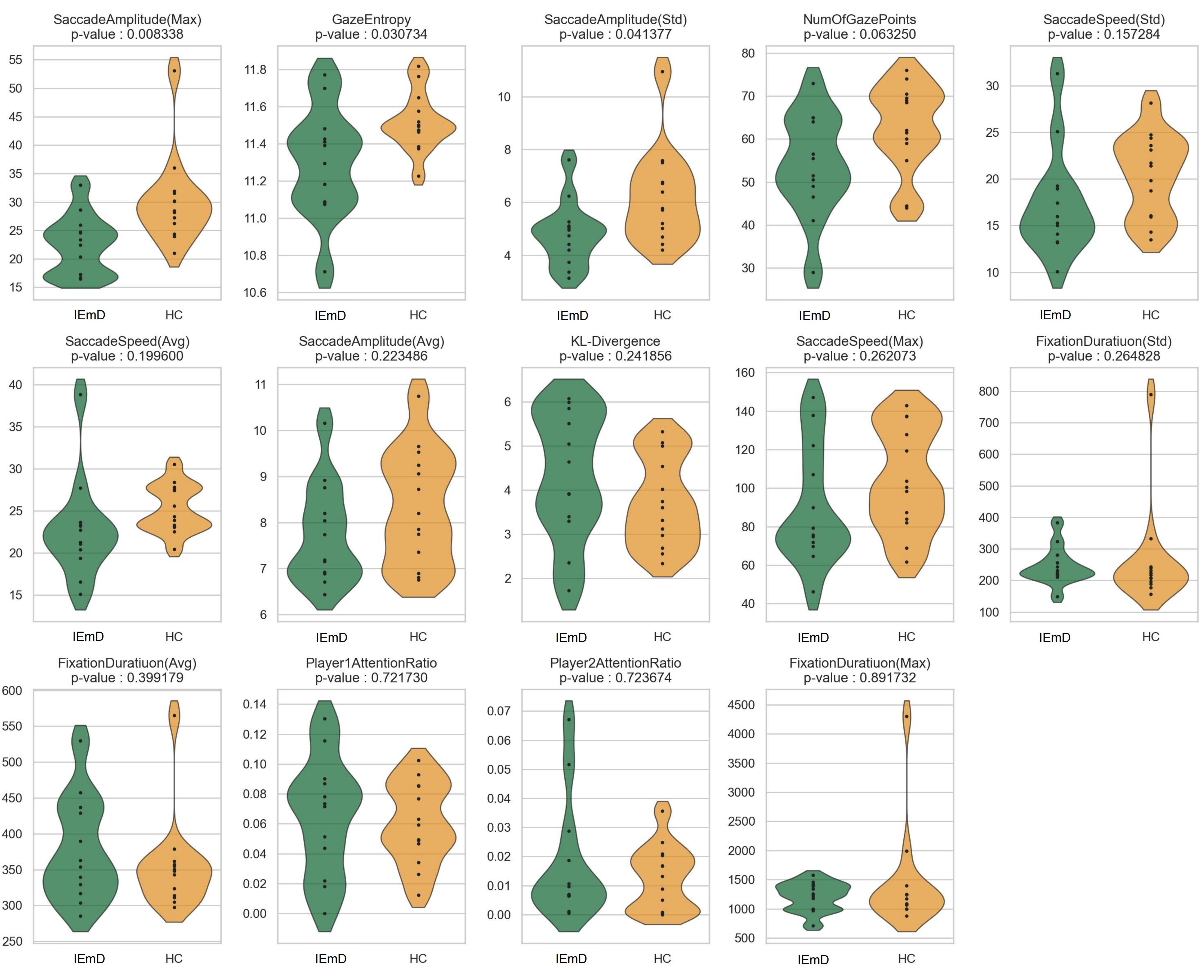

Supplement: Supplementary file 1 [file Image_1.jpg]

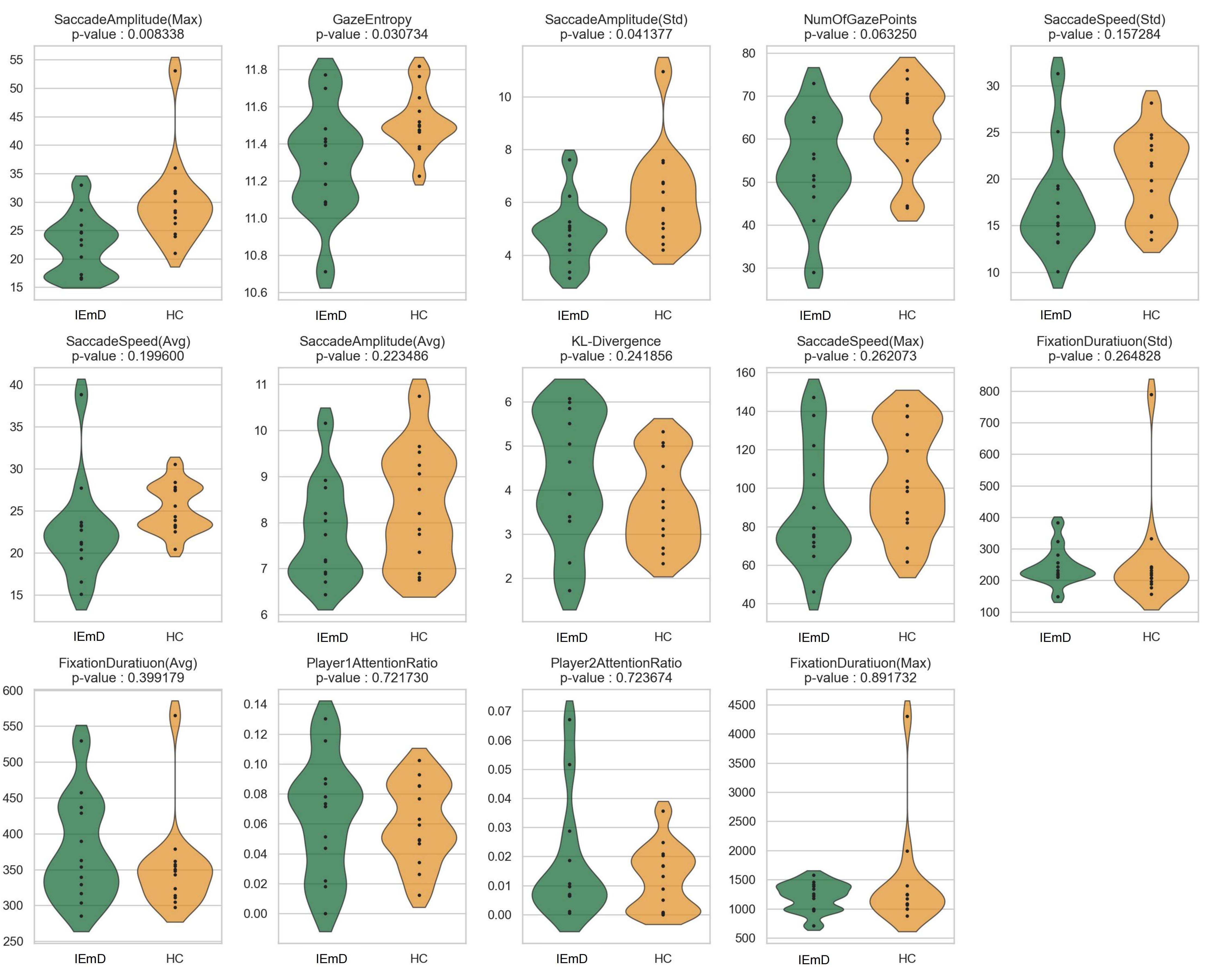

Supplement: Supplementary file 2 [file Image_2.jpg]
